# The Weight of Emotions: Childhood Obesity and Psychosocial Well-Being in Saudi Arabia

**DOI:** 10.3390/healthcare13172152

**Published:** 2025-08-29

**Authors:** Rabab Majzoub, Khalid Al Noaim, Abdulrahman Alnaim, Muneera Alabdulqader, Zainab Al Alawi, Sayed Ali, Abdulrazag Ibnshamsah, Abdulaziz Alanazi, Aljawhara Al Omair, Ahad Alaklabi, Kholud Alqhtani, Maha Alaklabi

**Affiliations:** 1 Pediatrics Department, College of Medicine, King Faisal University, Al-Ahsa 31982, Saudi Arabia; kalnoaim@kfu.edu.sa (K.A.N.); ahalnaim@kfu.edu.sa (A.A.); malabdulqader@kfu.edu.sa (M.A.); zalalwi@kfu.edu.sa (Z.A.A.); 2 Family and Community Medicine Department, College of Medicine, King Faisal University, Al-Ahsa 31982, Saudi Arabia; seali@kfu.edu.sa; 3 King Faisal University, Al-Ahsa 31982, Saudi Arabia; 220023308@student.kfu.edu.sa (A.I.); 220019997@student.kfu.edu.sa (A.A.); 220019491@student.kfu.edu.sa (A.A.O.); 4 King Khalid University, Abha 61421, Saudi Arabia; 439806187@kku.edu.sa (A.A.); 439806019@kku.edu.sa (K.A.); 5 College of Medicine, Bishah University, Bishah 61922, Saudi Arabia; 440802507@ub.edu.sa

**Keywords:** childhood obesity, psychological well-being, Saudi Arabia

## Abstract

Background/Objectives: Childhood obesity is a growing public health concern globally, with significant physical and psychological implications. While numerous studies have linked obesity to poor mental health outcomes, cultural differences may influence this relationship. This study examines the association between childhood obesity and psychological well-being among Saudi children aged 8–12 years. Methods: A cross-sectional case-control study was conducted in Al-Hofuf, Saudi Arabia. A total of 128 children were recruited, divided into a high-body-weight group (*n* = 64) and a healthy-weight group (*n* = 64). Psychological well-being was assessed using the Psychological Well-being Scale for Children (PWSC) and the Stirling Children’s Well-being Scale (SCWS). Anthropometric measurements were recorded, and parental body mass index (kg/m^2^) data were included. Data analysis was performed using SPSS 2023, employing chi-square tests and t-tests. Results: No statistically significant differences were found between the children’s body mass index (kg/m^2^) groups across most psychological well-being dimensions. However, a significant association was observed between maternal body mass index (kg/m^2^) and children’s PWSC scores (χ^2^ = 6.217, *p* = 0.045), suggesting a potential influence of parental weight on child well-being. Additionally, a significant sex difference in SCWS scores was noted, with males displaying higher well-being levels than females (χ^2^ = 7.798, *p* = 0.041). Family income and school grade showed no significant associations with psychological well-being. Conclusions: Contrary to findings from Western studies, childhood obesity did not significantly impact psychological well-being in this Saudi sample. Cultural factors, parental influence, and age-related differences may contribute to these results. Further longitudinal and qualitative research is needed to explore these associations in greater depth.

## 1. Introduction

Obesity is defined as an abnormal accumulation of body fat that adversely affects health, posing significant biomedical, economic, and psychosocial burdens [1,2]. According to the World Health Organization (WHO), the global prevalence of obesity has tripled since 1975. Childhood obesity has become a major health concern of the 21st century, affecting over 42 million children under five [3,4,5].

In the Kingdom of Saudi Arabia (KSA), childhood overweight and obesity rates were reported at 13.4% and 18.2%, respectively, with girls showing a slightly higher prevalence than boys [6].

Furthermore, the proportion of overweight children aged 4–12 increased from 5.4% in 2004 to 18.3% in 2016 [7]. According to the Saudi Ministry of Health, more than 15% of children under five were overweight, and 6% were obese, while among children aged five and older, 23% were overweight and 9.3% were obese [8].

Body mass index (BMI; kg/m^2^)–for-age percentile is commonly used to assess obesity in children, with sex-specific percentiles accounting for their ongoing growth. The Centers for Disease Control and prevention (CDC) categorized teen and child BMI (kg/m^2^) as follows: underweight as below the 5th percentile, healthy weight between the 5th and 85th percentiles, overweight as between the 85th and 95th percentiles, obesity as the 95th percentile or above, and severe obesity as a BMI (kg/m^2^) of 35 or higher [9].

Childhood obesity increases the risk of several medical complications, including diabetes mellitus, hypertension, coronary artery disease, stroke, gallbladder disease, osteoarthritis, sleep apnea, and hyperlipidaemia [1]. Beyond these physical consequences, obesity also severely impacts psychological well-being. Children with obesity are more likely to experience low self-esteem, anxiety, depression, and social isolation. They may face bullying, stigma, and body dissatisfaction, all of which contribute to emotional distress and hinder academic and social development [10,11,12].

A study in Egypt reported that 46.7% of children with obesity experienced bullying, which led to emotional difficulties and poor self-worth. Childhood Depression Inventory scores were positively correlated with BMI (kg/m^2^) and waist circumference, and 26.7% of children with obesity had severely low self-esteem, compared to just 10% of their normal-weight peers [13].

Obesity negatively affects body image and children’s well-being [14]. A study conducted in Mexico, found that 74% of children were dissatisfied with their body image, with overweight or children with obesity being 6.73 times more likely to experience body dissatisfaction than their normal-weight counterparts [15].

Cognitive function may also be affected by obesity. Studies have shown a decline in working memory and other cognitive scores as BMI (kg/m^2^) increases. For instance, children with obesity in a Malaysian study scored, on average, 4.075 points lower than their normal-weight peers in cognitive assessment [11,16]. Psychological distress can create a vicious cycle, leading to poor eating habits and reduced physical activity, further worsening obesity and associated mental health challenges [17,18,19].

Socioeconomic status (SES) is another key determinant. Numerous studies have shown that children from lower SEC backgrounds face a greater risk of obesity due to limited access to healthy food, safe physical activity environments, and exposure to chronic stress [20,21,22,23,24,25].

A 2022 systematic review examining Mexican children found that those with overweight or obesity, particularly girls, were more susceptible to depression [26].

In 2016, a study from Western Saudi Arabia reported that higher BMI (kg/m^2^) in children was associated with physical limitations, attention difficulties at school, and social challenges. Interestingly, no strong correlation was found with emotional issues or absenteeism [27]. The psychological impact of obesity is not limited to childhood: a 2023 cross-sectional study in Riyadh found that one-third of adults with obesity also experienced increased levels of anxiety and depression [28].

Due to the limited number of studies exploring the psychological implications of childhood obesity in the Saudi context, this study was conducted. The rapid increase in the prevalence of childhood overweight and obesity over the past two decades in Saudi Arabia raises concerns about both physical and psychological consequences [29]. Unlike many prior studies—such as those conducted by Blanco et al. and Putra et al—which relied on self -administered questionnaires, this research employed structured face-to-face interviews. This approach reduces potential biases related to question misinterpretation and ensures more accurate data collection [30,31].

This paper aims to investigate the relationship between childhood overweight and obesity and various parameters of psychological well-being, with a focus on determining whether this association is predominantly positive or negative. While global studies on childhood obesity largely focus on Western contexts, the unique cultural and family-related influences in Saudi Arabia remain underexplored, particularly the role of parental obesity in shaping children’s psychological well-being. This study addresses this gap by examining the association between parental BMI (kg/m^2^) and the psychological health of children with obesity.

## 2. Materials and Methods

### 2.1. Study Design

This study followed a hybrid cross-sectional case-control design to explore the relationship between childhood obesity and psychological well-being. The research was conducted in Al-Hofuf, Saudi Arabia, King Faisal University’s (KFU) health center from January 2024 to June 2024. The study population consisted of children aged 8 to 12 years, divided into two groups that are the high-body-weight group with a higher (kg/m^2^) than normal (case group) and the healthy-weight group with a normal BMI (kg/m^2^) (control group), based on CDC classification for children BMI [9]. The required sample size was determined using G*Power 3.1.9.2, based on a power analysis for a two-sample t-test, considering an effect size (Cohen’s d) of 0.50, a significance level (α) of 0.05, and a power (1-β) of 0.80, resulting in a total of 128 participants (64 per group).

### 2.2. Data Collection

Participants were carefully matched on key demographics (e.g., SES, age, sex) according to specific criteria to recruit children of both sex, aged 8 to 12 years, and attending King Faisal University’s (KFU) health center to receive health care, in which children who have participated were distributed between the healthy-weight and high-body-weight groups according to their BMI. Additionally, informed consent from legal guardians was obtained before participation. Children were excluded if they were outside the age range, non-Arabic speakers, or had neurological disorders affecting cognition, psychological disorders requiring treatment, or obesity secondary to pharmacological treatment. Children receiving endocrinological treatment for obesity or females with early menarche were also excluded.

The questionnaire Supplementary File employed in the study consisted of biographical data alongside 24 questions aimed at assessing the psychological and emotional states of children. To achieve this, two validated psychological well-being scales were incorporated. The Psychological Well-being Scale for Children (PWS-C), developed by Opree and colleagues in 2018, served as a key instrument in evaluating children’s emotional health [32]. Similarly, the Stirling Children’s Well-being Scale (SCWBS), formulated by Liddle and Carter in 2015, offered additional insights into the overall psychological well-being of the participants. Each of these scales contained 12 items, contributing to a holistic assessment of children’s emotional and psychological states [33].

Beyond psychological evaluation, the study also integrated anthropometric measurements to explore potential links between physical attributes and psychological well-being. Data on height (cm), weight (kg), and BMI (kg/m^2^) were collected for each child, while parental height (cm), weight (kg), and BMI (kg/m^2^) were also recorded to provide a broader perspective on familial influences.

### 2.3. Validity and Reliability Testing

To ensure the validity and reliability of the English-to-Arabic questionnaire used for data collection, a pilot study and back-translation validation process were conducted with 26 children receiving care at King Faisal University Polyclinic. Well-trained medical students assisted children in completing the questionnaire. Once the questionnaire was deemed valid and reliable, it was utilized for the main study. The primary method of data collection involved trained data collectors administering a Google Forms questionnaire to gather information on psychological well-being, obesity assessment according to CDC, and demographic data (age, sex, and family income).

This study utilized a structured approach to data collection from the 128 participants, whose legal guardians agreed to participate via face-to-face interviews between data collectors and children who met the predefined inclusion and exclusion criteria alongside their caregivers.

### 2.4. Statistical Analysis

For statistical analysis, data were processed using SPSS 2023 where numerical data in the study were presented as mean and standard deviation (SD), while categorical data were expressed as percentages and frequencies. To analyse differences in psychological well-being between children with high body weight and those with a healthy weight, the Kolmogorov–Smirnov test was carried out along with visual inspection of histograms and Q–Q plots to test for normality of the outcome variables and the data showed normal distribution. Therefore, t-tests were used for numerical comparisons and chi-square tests for categorical associations. A *p* value of <0.05 for 95 confidence interval was considered significant.

## 3. Results

### 3.1. Demographic Variables

The demographic details of the participants can be seen in Table 1 with the main focus being on the categorical variables such as sex, school grade, family income. In addition, (kg/m^2^) values for children and parents are provided as well. Most of the participants were males (60.2%), and the Grade 6 (32.0%) was the most common school grade among children in both groups.

Regarding socioeconomic status, family income was categorized into three groups: low income (<SAR 8988/<USD 2427), comprising 26.6% of participants; moderate income (SAR 8988–14,980/USD 2427–4045), accounting for 32.8%; and high income (>SAR 14,980 SR/>USD 4045), which represented the largest proportion (40.6%). Upon assessment of children’s BMI (kg/m^2^) values, this helped to distribute participants between two groups of healthy weight (50.8%) and high body weight (49.2%). BMI (kg/m^2^) was calculated as well for parents giving the piece of knowledge that most fathers (78.9%) and mothers (70.3%) were categorized as having high body weight. Participants had a mean age of 10.05 years, with an (SD) of 1.5; the age range was from 8 to 12 years.

### 3.2. Psychological Well-Being Scale for Children (PWSC)

Table 2, below, presents the results of the PWSC scale, which measures various dimensions of psychological well-being in children. The t-values, degrees of freedom (df), and *p*-values are reported for each item. A *p*-value < 0.05 indicates statistical significance.

None of the individual items or the total PWSC score reached statistical significance (*p* > 0.05). This suggests that, in this sample, there were no significant differences or trends in psychological well-being between children with a high body weight and those of normal weight, as measured by the PWSC scale.

### 3.3. Stirling Children’s Well-Being Scale (SCWS)

Like the PWSC, Table 3 shows that none of the individual items or the total SCWS score reached statistical significance (*p* > 0.05).

This indicates that, in this sample, there were no significant differences or trends in the positive emotional states or outlooks between children with high body weight and those of normal weight, as measured by the SCWS scale.

### 3.4. PWSC and SCWS

Table 4 presents the distribution of scores for two psychological well-being scales, categorized into three levels as low, moderate, and high: the PWSC and the SCWS. Both scales show a similar pattern of responses, with the majority of children falling into the “moderate” category (52.3% for PWSC and 51.6% for SCWS). The “low” category accounts for 25.0% of participants on both scales, while the “high” category represents 22.7% for PWSC and 23.4% for SCWS. This similarity suggests consistency in the measures of well-being between the two scales within this sample.

### 3.5. Demographic Variables and PWSC

Table 5 presents the chi-square test results examining the association between various demographic variables and the PWSC. The findings indicate no significant associations between PWSC and sex (χ^2^ = 1.874, *p* = 0.392), school grade (χ^2^ = 14.236, *p* = 0.432), or family income (χ^2^ = 5.771, *p* = 0.673). However, a statistically significant relationship is observed between the maternal BMI (kg/m^2^) and PWSC scores (χ^2^ = 6.217, *p* = 0.045), suggesting that children of mothers with high BMI (kg/m^2^) are more likely to have increased well-being scores.

### 3.6. Demographic Variables and SCWS

Table 6 reports the results of chi-square tests evaluating the associations between demographic variables and the SCWS. A significant relationship is observed between sex and SCWS scores (χ^2^ = 7.798, *p* = 0.041), with males showing a higher proportion in the “high” well-being category. Additionally, significant associations are found between SCWS scores and the BMI (kg/m^2^) of both fathers (χ^2^ = 6.059, *p* = 0.048) and mothers (χ^2^ = 8.638, *p* = 0.013), with children of parents in the “high body weight” category exhibiting higher well-being scores. Conversely, no significant associations are noted for school grade (χ^2^ = 5.763, *p* = 0.972) or family income (χ^2^ = 3.4, *p* = 0.907).

### 3.7. Overall Interpretation

There were no statistically significant data upon analysis of children’s psychological well-being, positive emotional state and positive outlook by the two scales used in the mean of PWSC and SCWS.

In the final analysis, the psychological well-being, positive emotional states and positive outlook of the participants did not vary significantly between the healthy-weight and high-body-weight groups.

## 4. Discussion

The overall aim of the current study was to examine the relationship between childhood obesity and psychological well-being in Saudi children aged 8–12 years.

Contrary to results of earlier studies such as those conducted by Topçu et al. and Lindberg et al., our analysis revealed no statistically significant differences between overweight/obese and normal-weight children for the majority of the psychological well-being dimensions of the (PWSC) and the (SCWS) [34,35].

Previous studies have frequently reported high associations between childhood obesity and worse psychological health to suggest heightened vulnerability to anxiety, depression, and lower self-esteem in obese and overweight children [13,14,15], like the study titled “Psychological Aspects of Obesity in Children and Adolescents” that was published in 2018, have extensively reported the relationship between childhood obesity and psychosocial well-being [36].

In addition, a more recent European study, in 2023, found that higher BMI (kg/m^2^) in children was negatively associated with psychological and emotional health to suggest a direct adverse influence of obesity on psychological outcomes [37].

Similarly, a Spanish study revealed significantly lower self-esteem and heightened teasing in children with obesity compared to their peers of normal weight, thus emphasizing psychological burdens linked to excess weight. However, our findings did not align with these results, prompting further examination of possible explanations for this divergence [30].

One explanation for our non-significant findings could be cultural differences. Living in an Islamic society, strictly following Islamic values which prohibit bulling is one of the important factors that impact the perception of self-image that might also be implicated. Saudi Arabia’s social and cultural dynamics might differ from Western populations, potentially providing protective psychological effects against weight stigma [38].

More tolerance for body size differences among children within cultures, as well as stronger support within families, may counteract or mask the psychological distress usually associated with obesity [39].

Our age group of participants (8–12 years) could also be the reason behind these non-significant results. Psychological disturbances related to obesity might become apparent at the adolescence stage, a period that is defined by heightened sensitivity towards self-esteem, peer acceptance, and social comparison [40]. Younger children within our sample may have yet to experience the same level of psychological distress as compared to older age groups.

Longitudinal studies that follow children over time as they grow into adolescence would be helpful in determining the potential developing relationship between obesity and psychological well-being. Interestingly, our study found strong correlation between maternal BMI (kg/m^2^) and children’s psychology scores on the PWSC (χ^2^ = 6.217, *p* = 0.045). Specifically, children of mothers with higher BMI (kg/m^2^) reported differences in psychological well-being outcomes.

This is also aligned with current evidence that maternal health and household environment can significantly influence children’s emotional and behavioral development. Similar results were revealed by the previous European research that credited family BMI (kg/m^2^) and way of life with the mental health of children and accentuated the indirect influence of the family background and lifestyle characteristics on children’s emotional well-being [37].

We also obtained a statistically significant sex difference using the SCWS where males reported higher percentages in the “high” well-being group than females (χ^2^ = 7.798, *p* = 0.041).

This sex difference could reflect culturally conditioned social pressures or expectations for body image and behavior, which might differently affect girls and boys.

Additional research is recommended to explore these sex-specific psychological consequences more fully in local cultural contexts.

The absence of significant correlations with family income and educational level (grade at school) suggests that these socio-economic and educational determinants are possibly not strong predictors of children’s psychological well-being.

This is set against typical assumptions about socioeconomic impacts elsewhere and it shows the complexity and potential interaction of several determinants acting on psychological well-being regardless of economic status or grade at school.

Conflict in findings is present according to differences in study design, sample populations, and methodological approaches.

The key point drawn by previous research is that while obesity usually occurs with psychiatric struggles, not all children with obesity are faced with meaningful psychopathology. A couple of researchers found high correlations between obesity and psychopathology [34,35] whereas others did not document a direct association between these phenomena [41,42].

Additional variables such as sex, age, race, and obesity magnitude have been contended to be potential regulators of this relationship, highlighting the complexity of obesity’s psychological effects.

Moreover, our findings reiterate that obesity is associated with an increased threat of mental maladjustment in the form of depressive symptoms, anxiety, and self-esteem problems. In addition, past research highlighted the role played by family and social context, such as parenting styles, peer victimization, and social support in the psychological well-being of children with obesity [43,44,45]. Our findings also emphasize the agency of these factors, validating the implication that social stressors play a central role in modulating the mental health effects of obesity. Similarly, existing studies show that women with obesity are disproportionately affected by psychological distress, particularly in terms of body image concerns [46,47]. Our findings are consistent with this perspective, indicating that sex exerts a powerful influence on the psychological impact of obesity such that girls are more distressed than boys.

This study applies standard, validated measurement tools to provide more consistent, better results. In addition, our research mixes both clinical samples and population samples, providing a larger perspective on the psychological effects of obesity in diverse settings. Empirical research regarding childhood obesity and mental health is still not commonly present in some parts of the world, particularly in South Asia and the Middle East. The current research fills the gap to some extent by concentrating on various populations, hence providing cross-cultural data on the psychological effects of obesity.

Our study builds upon previous research by integrating additional variables, such as parental BMI, as potential determinants of childhood psychological well-being.

Notably, Puhl and Brownell conducted a study that explored the impact of weight-based stigma on psychological health in overweight adults and adults with obesity, concluding that stigma alone was not directly predictive of mental health outcomes; rather, coping mechanisms played a mediating role. In contrast, our study shifts the focus to a younger population and examines psychological well-being without assuming stigma as an intermediary factor [48].

In addition, Magallares and Pais-Ribeiro conducted a meta-analysis that questioned the assumption that obesity universally correlates with poor mental health, suggesting that some individuals—particularly men—exhibit better psychological well-being despite high BMI, a phenomenon described as the “Jolly Fat” hypothesis. Our study extends this perspective by demonstrating that children with higher-weight parents tend to report better well-being scores, suggesting that familial factors may modulate psychological responses to obesity [49].

Key advancements over prior research are also evident in our study. Whereas most previous studies have examined adults, our study provides a novel contribution by focusing on children, thereby elucidating the early-life influences of obesity on psychological well-being. Unlike studies that primarily assess individual BMI, our research highlights the role of parental BMI (kg/m^2^) in shaping children’s psychological outcomes, suggesting an intergenerational transmission of psychological resilience related to weight status. Our study incorporates direct statistical comparisons using validated psychological well-being scales specifically designed for pediatric populations. In contrast to earlier research that has largely overlooked demographic moderators, our study integrates variables such as sex, school grade, and family income, enabling a more nuanced analysis of potential influencing factors. Our findings indicate that boys report higher psychological well-being than girls, adding a sex-based dimension to the discourse and providing new hypotheses regarding sociocultural influences on obesity and mental health.

### 4.1. Implications for Future Research

The findings of our study open several avenues for further investigation. Future studies should include larger sample sizes and mixed methods to capture complex psychological impacts and better grasp participants’ emotional experiences.

The future research should explore the role of parental attitudes, health behaviors, and psychosocial support as mediators in the association between parental BMI (kg/m^2^) and child well-being. Longitudinal studies are needed to assess whether the observed associations persist into adolescence and adulthood, providing deeper insights into the long-term psychological consequences of childhood obesity. Given the complex interplay between obesity and mental health, incorporating additional psychological constructs such as resilience, self-efficacy, and social support may enhance our understanding of the protective and risk factors involved. Further exploration of cultural and socioeconomic moderators is warranted to determine whether the observed relationships hold across diverse populations and socioeconomic strata.

### 4.2. Study Limitations

There are several limitations to this study. As a cross-sectional case-control study, it cannot assess causal relationships as the exposure and outcome data were collected at the same time, making it impossible to determine whether the exposure preceded the outcome, or was a result of the outcome. Also, recall bias could muddle responses to self-reported psychological and behavioral measures. Additionally, the participant bias measures in the study limit the findings if the cases and controls did not adequately represent the greater population. Furthermore, the design did not look at incidence or risk, but at prevalence of possibilities, limiting extrapolating findings about the progression of psychological outcomes over time. There may have also been unmeasured confounding variables regarding the relationships we made conclusions about.

Besides the methodological challenges, this study did not measure parental psychological well-being, which is a significant parent contributor to children’s mental health. Also, certain cultural aspects that would pertain to Saudi Arabian residents could have a bearing on how the perception of, and experiences of obesity and psychological distress are accounted for, which would affect generalizability. While there was an association between maternal BMI (kg/m^2^) and children’s well-being, underlying factors in the association were not measured, which could include parenting style, emotional dynamics within the household, or shared lifestyle behaviors. Lastly, the young age groups could not be represented adequately in the sample and may limit the generalizability for a younger child age group.

## 5. Conclusions

In conclusion, this study indicates that, within the Saudi context, obesity alone might not be a predominant determinant of psychological well-being in children aged 8–12. Cultural, familial, and environmental factors, such as maternal health, likely exert more substantial impacts. Longitudinal studies and culturally tailored qualitative research are recommended to better understand these complex interactions and to confirm the generalizability of these findings within broader Saudi and regional populations.

## Figures and Tables

**Table 1:** Demographic variables of participants (*n* = 128).

Demographic Variable		*n*	%
Sex	Male	77	60.20
	Female	51	39.80
Age (years)	Mean	SD	Min–Max
	10.05	1.5	8–12
School grade	1st	7	5.47
	2nd	17	13.28
	3rd	24	18.75
	4th	24	18.75
	5th	15	11.72
	6th	41	32.03
Family income	Low (<SAR 8988/<USD 2427)	34	26.60
	Moderate (SAR 8988–14,980/USD 2427–4045)	42	32.80
	High (>SAR 14,980/>USD 4045)	52	40.60
BMI (kg/m^2^) child	High body weight	63	49.20
	Healthy weight	65	50.80
Paternal BMI (kg/m^2^)	Normal	28	21.80
	Overweight	59	46.10
	Obese I	31	24.20
	Obese II	7	5.40
	Obese III	3	2.50
Maternal BMI (kg/m^2^)	Normal	27	21.10
	Overweight	45	35.20
	Obese I	30	23.40
	Obese II	18	14.10
	Obese III	8	6.20

**Table 2:** Questions assessing the six aspects of psychological well-being.

Psychological Well-Being Scale for Children (PWSC)	t	df	*p*
Environmental mastery: do you yourself choose what you do after school?	0.2	126	0.841
Environmental mastery: do you yourself choose what you do at the weekend?	0.543	126	0.587
Personal growth: do you like to engage in new activities?	−0.572	126	0.568
Personal growth: do you like meeting new people?	1.389	126	0.167
Purpose in life: do you think about what you want to be when you grow up?	−0.203	126	0.839
Purpose in life: do you think about where you want to live in the future?	−0.443	126	0.658
Self-acceptance: are you happy with yourself?	1.272	126	0.206
Self-acceptance: are you satisfied with who you are?	0.151	126	0.88
Autonomy: do you ask your parents for their opinion?	1.113	126	0.267
Autonomy: do you ask your parents for help?	1.143	126	0.255
Positive relations: do you do fun things with your friends?	−0.425	126	0.671
Positive relations: do you do fun things with your parents?	0.007	126	0.994
Total PWS	0.648	126	0.518

**Table 3:** Questions assessing positive emotional state and positive outlook.

Stirling Children’s Well-Being Scale (SCWS)	t	df	*p*
Positive emotional state: I’ve been feeling calm	−0.648	126	0.518
Positive emotional state: I’ve been feeling cheerful about things	1.323	126	0.188
Positive emotional state: I’ve been feeling relaxed	1.413	126	0.16
Positive emotional state: I’ve been in a good mood	0.896	126	0.372
Positive emotional state: I’ve been getting on well with people	−0.416	126	0.678
Positive emotional state: I enjoy what each new day brings	−1.029	126	0.305
Positive outlook: I think there are many things that I can be proud of	−1.703	126	0.091
Positive outlook: I feel that I am good at some things	−1.353	126	0.178
Positive outlook: I think good things will happen in my life	−1.448	126	0.15
Positive outlook: I can find lots of fun things to do	0.45	126	0.653
Positive outlook: I think lots of people care for me	−0.567	126	0.571
Positive outlook: I’ve been able to make choices easily	−1.921	126	0.057
Total SCWPS	−0.641	126	0.522

**Table 4:** Distribution of scores for the two scales (PWSC and SCWS).

Scale	Category	Score Range	*n*	%
PWSC	Low	≤33.5	32	25.0
	Moderate	Between 33.5 and 43	67	52.3
	High	>43	29	22.7
SCWS	Low	≤45.5	32	25.0
	Moderate	Between 45.5 and 57	66	51.6
	High	>57	30	23.4

**Table 5:** Chi-square test results examining the association between demographic variables and PWSC.

	Chi-Square	*p*-Value
Sex VS WSC	1.874	0.392
School grade VS PWSC	14.236	0.432
Family income VS PWSC	5.771	0.673
Paternal BMI (kg/m^2^) VS PWSC	8.296	0.031
Maternal BMI (kg/m^2^) VS PWSC	6.217	0.045

**Table 6:** Chi-square test evaluating the associations between demographic variables and SCWS.

	Chi-Square	*p*-Value
Sex VS SCWS	7.798	0.04
School grade VS SCWS	5.763	0.97
Family income VS SCWS	3.4	0.90
Paternal BMI (kg/m^2^) VS SCWS	6.059	0.04
Maternal BMI (kg/m^2^) VS SCWS	8.638	0.01

## Data Availability

The datasets used and analyzed during the current study are available from the corresponding author upon reasonable request.

## Supplementary Materials

The following supporting information can be downloaded at: https://www.mdpi.com/article/10.3390/healthcare13172152/s1, the questionnaire of the study.

## Abbreviations

The following abbreviations are used in this manuscript:

PWSC	Psychological Well-being Scale for Children
SCWS	Stirling Children’s Well-being Scale
BMI	Body mass index
SES	Socioeconomic status
