# Supplementary material for: The Weight of Emotions: Childhood Obesity and Psychosocial Well-Being in Saudi Arabia
Source: Healthcare (Basel). 2025 Aug 29;13(17):2152. doi: 10.3390/healthcare13172152 (PMC12427818; doi:10.3390/healthcare13172152)
Supplement: Supplementary file 1 [file healthcare-13-02152-s001.zip › healthcare-3731329-supplementary.pdf]

The Weight of Emotions: Childhood Obesity and Psychosocial Well-Being in Saudi Arabia

A study aimed at determining the extent of the impact of obesity on children's mental health

All information provided will be treated confidentially for scientific research purposes

1. **Question for the child's legal guardian:**
   Do you agree to the participation of the child under your responsibility in this study?
   - Agree
   - Disagree
2. **Section: Social and Demographic Data**
3. **Sex :**
   - Male
   - Female
4. **Age:**
   - 8
   - 9
   - 10
   - 11
   - 12
5. **Nationality:**
   - Saudi
   - Other (please specify)
6. **Place of residence:**
   - Al-Ahsa
   - Dammam
   - Khobar
   - Hafar Al-Batin
   - Jubail
   - Qatif
   - Other (please specify)
7. **Grade (school year):**
   - 1st grade (primary)
   - 2nd grade
   - 3rd grade
   - 4th grade
   - 5th grade
   - 6th grade
   - Other

8. **Family monthly income:**
    - Less than 5,000 SAR
    - 5,000 – 10,000 SAR
    - 10,000 – 15,000 SAR
    - 15,000 – 20,000 SAR
    - More than 20,000 SAR

Section: Children's Mental Health Scale

Psychological wellbeing via psychological wellbeing scale for children (PWS-c)

9. (Environmental mastery) Do you choose activities after school by yourself or are you told what to do?
    - Rarely
    - Sometimes
    - Often
    - Very often
10. (Environmental mastery) Do you choose your weekend activities yourself, or are you told what to do?
    - Rarely
    - Sometimes
    - Often
    - Very often
11. (personal growth)Do you like to participate in new activities?
    - Rarely
    - Sometimes
    - Often
    - Very often
12. (personal growth)Do you like to meet new people?
    - Rarely
    - Sometimes
    - Often
    - Very often
13. (purpose in life) Do you think about what you want to become when you grow up?
    - Rarely
    - Sometimes
    - Often
    - Very often
14. (purpose in life) Do you think about where you want to live in the future?
    - Rarely
    - Sometimes
    - Often

- Very often

15. (Self-acceptance)Are you happy with yourself?
    - Rarely
    - Sometimes
    - Often
    - Very often
16. (Self-acceptance)Are you satisfied with who you are?
    - Rarely
    - Sometimes
    - Often
    - Very often
17. (Auronomy) Do you ask your parents for their opinions?
    - Rarely
    - Sometimes
    - Often
    - Very often
18. (Auronomy) Do you ask your parents for help in some tasks?
    - Rarely
    - Sometimes
    - Often
    - Very often
19. (Positive relations) Do you do fun activities with your friends?
    - Rarely
    - Sometimes
    - Often
    - Very often
20. (Positive relations) Do you do fun activities with your parents?
    - Rarely
    - Sometimes
    - Often
    - Very often

Section: Stirling Children's Wellbeing Scale

21. (Positive emotional state) At the present time, do you feel calm?
    - Never
    - Rarely
    - Sometimes
    - Often
    - All the time
22. (Positive emotional state) At the present time, do you feel joy about things around you?
    - Never
    - Rarely
    - Sometimes

- Often
- All the time

23. (Positive emotional state) At the present time, do you feel relaxed?
    - Never
    - Rarely
    - Sometimes
    - Often
    - All the time
24. (Positive emotional state) At the present time, are you in a good mood?
    - Never
    - Rarely
    - Sometimes
    - Often
    - All the time
25. (Positive emotional state) At the present time, do you interact well with people?
    - Never
    - Rarely
    - Sometimes
    - Often
    - All the time
26. (Positive emotional state) At the present time, do you enjoy new things you experience during the day?
    - Never
    - Rarely
    - Sometimes
    - Often
    - All the time
27. (Positive outlook) Do you think there are many things in your life to be proud of?
    - Never
    - Rarely
    - Sometimes
    - Often
    - All the time
28. (Positive outlook) Do you think you are good at some things?
    - Never
    - Rarely
    - Sometimes
    - Often
    - All the time
29. (Positive outlook) Do you think good things will happen in your life?
    - Never
    - Rarely
    - Sometimes
    - Often
    - All the time
30. (Positive outlook) Do you find many fun things to do?

- Never
- Rarely
- Sometimes
- Often
- All the time

31. (Positive outlook) Do you think many people care about you?
    - Never
    - Rarely
    - Sometimes
    - Often
    - All the time
32. (Positive outlook) Are you able to make decisions easily?
    - Never
    - Rarely
    - Sometimes
    - Often
    - All the time

Section: Anthropometric and Family Data

33. Are there psychological or social problems in your family?
    - Yes
    - No
    - Other (specify)
34. Has there been weight loss or weight gain in the child in the last six months?
    - Yes
    - No
    - Other (specify)
35. Child's height (cm)
36. Child's weight (kg)
37. Child's BMI ((kg/m^2) %
38. Child's waist circumference (cm)
39. Father's height (cm)
40. Father's weight (kg)
41. Father's BMI ((kg/m^2)%
42. Mother's height (cm)
43. Mother's weight (kg)
44. Mother's BMI ((kg/m^2)%
